# Effect of co-application of phosphorus fertilizer and *in vitro*-produced mycorrhizal fungal inoculants on yield and leaf nutrient concentration of cassava

**DOI:** 10.1371/journal.pone.0218969

**Published:** 2019-06-26

**Authors:** Ibrahim A. Aliyu, Ado A. Yusuf, Edward O. Uyovbisere, Cargele Masso, Ian R. Sanders

**Affiliations:** 1 Department of Soil Science, Ahmadu Bello University, Zaria, Nigeria; 2 International Institute of Tropical Agriculture, Yaoundé, Cameroon; 3 Department of Ecology and Evolution, Université de Lausanne, Lausanne, Switzerland; University of California Berkeley, UNITED STATES

## Abstract

The adaptability of cassava to low fertile and marginal soils facilitates its production in subsistent agriculture. As a result, smallholder farmers rarely apply fertilizers. The current yield gap is therefore very large, calling for application of fertilizers and soil amendments to improve its productivity. Field experiments were carried out to assess the potential of partially substituting Phosphorus (P) fertilizers by *in vitro*-produced arbuscular mycorrhizal fungal (AMF) inoculants in cassava production in two agro-ecologies of Nigeria: Northern Guinea Savanna (Samaru) and Sudan Savanna (Minjibir). The experiments were laid out in a split plot design with P levels (0, 17.5, 35 and 52.5 kg P_2_O_5_ ha^-1^) as main plot and AMF inoculants (Control, Glomygel, Glomygel carrier, Mycodrip, Mycodrip carrier) as subplots. The results in Samaru showed that there was significant interaction between AMF and P in root fresh weight, total biomass and root to shoot ratio. The root fresh weights of the inoculated cassava increased proportionally with application of P. However, highest root fresh weight of cassava inoculated with Glomygel was observed at 35 kg P_2_O_5_ ha^-1^ recording 25% yield increase compared to 52.5 kg P_2_O_5_ ha^-1^ application. Interestingly, Cassava inoculated with Glomygel at 17.5 kg P_2_O_5_ ha^-1^ gave root fresh yield statistically similar to where 35 kg P_2_O_5_ ha^-1^ was applied. This represented a 50% reduction in P fertilizer use. Also, cassava inoculated with Glomygel increased leaf nutrient concentrations, which strongly correlated with the root fresh yield. However, no effects of inoculant carriers were observed in yield and nutrient concentrations. Contrarily, there was no significant treatment effect in Minjibir for nearly all the measured parameters. Cassava yield was however, higher in Minjibir than Samaru probably due to soil fertility and structural differences, which resulted in few observable effects of AMF and P treatments at Minjibir. We conclude that under low P conditions inoculation with *in vitro* produced AMF inoculants could be employed to reduce P fertilizer requirements for cassava and improve yields, but the variability of the responses as a result of soil heterogeneity and the identity of the fungal strain in the inoculant require further investigations before recommending the practice.

## Introduction

Phosphorus (P) is one of the most important determinants of plant growth and in most soils it exists in forms that are largely unavailable for plant uptake [1]. Such is the case with Nigerian soils, which are mostly Oxisols, Ultisols and Alfisols [2]. Phosphorus can be as low as 2 mg kg^-1^ in the savanna soils of Nigeria [3] thus, making P one of the most limiting nutrients in those soils. Kamara et al. [4] reported that P levels were lower than critical values of 7 mg kg^-1^ (Mehlich-3 extractable P) in 92% and 93% of the fields surveyed in the Northern Guinea savanna and the Sudan savanna of Nigeria, respectively. Overcoming P deficiency in soils of savanna zones of Nigeria is one of the major challenges that smallholder farmers are facing. Therefore, improved P acquisition is essential in order to improve crop yields in extremely low-P conditions [5]. However, inorganic P fertilizers are not easily affordable for resource-constrained farmers who represent the majority of the farming community in sub-Saharan Africa. As a consequence, most farmers often apply rates of P that are too low to obtain a significant yield response. Hence, there is a need to provide cost-effective alternatives in order to increase the adoption of high quality agricultural inputs by reducing the cost of production, while enhancing crop productivity.

Some soil microbial inoculants could serve as possible alternatives as they offer the potential to increase agricultural yields and productivity in low-input systems [6]. Research is revealing the various mechanisms by which soil microbes can stimulate plant productivity [7]. In particular, symbionts of plant roots, namely arbuscular mycorrhizal fungi (AMF), have received considerable attention as a potential low-input solution to increasing the nutrient uptake efficiency of crops. The majority of plant species, including most agricultural crops, enter into symbioses with AMF, exchanging plant sugars for fungal-derived nutrients, such as phosphorus and nitrogen [8]. They increase plant growth and nutrient uptake and can improve plant tolerance to root pathogens and drought [9,10,11]. It has been demonstrated that plants can receive up to 100% of their P via the mycorrhizal pathway, and from 4 to 20% of plant carbon can be transferred to the fungi [12].

Cassava (*manihot esculenta* Crantz) is reported to be highly tolerant to soils with low levels of P [13]. This is because it can tolerate highly leached soils low in pH, high in exchangeable Al [14]. On the other hand, cassava forms a mutually beneficial association with AMF [15], which allows cassava to absorb sufficient P for healthy growth. Inoculation with AMF can, therefore, lead to growth increases in cassava [16]. According to report by [17], AMF inoculation of cassava can increase fresh root weight by up to 5 t ha^-1^. There are also several studies in Nigeria that have shown that cassava roots establish associations with AMF [18], while others demonstrated the impact of AMF on cassava growth [19,20]. These studies evidently showed that AMF could play an important role in increasing the sustainability of cassava cropping in Nigeria. However, the inoculants used in these studies were produced in large amounts, requiring much labor, time and space [18,21]. In most cases (e.g. [22]) the inoculum was soil-based, where a suitable trap crop is used to generate AMF propagules, which are produced in the soil. The soil is then used as inoculant. Such inoculants may be low in propagule number and contaminated with other potentially undesirable microorganisms. Another disadvantage of such inoculants is that they may be difficult to transport because of the large volume of the substrate required.

A biotechnological system of AMF production through *in vitro* cultivation offers unique advantage in producing AMF in sterile medium. Some small enterprises have developed efficient production systems allowing the concentration of very large numbers of AMF propagules in a small volume of sterile medium. This makes the product easy to transport, free of unwanted microorganisms and potentially economically viable for large-scale application [23]. Other advantages of *in vitro* AMF production technique include more precise quality control such as non-destructive microscopic observations of cultures, uncontaminated molecular analyses and mass production at an industrial scale. An example of *in vitro* produced AMF is *Rhizophagus irregularis*, which can be produced at a large scale in a sterile medium. In a field experiment, Ceballos et al. [24] reported that *in vitro* produced *R*. *irregularis* significantly increased cassava yields in Colombian soils by approximately 20% compared to the non-inoculated control. Hence, it is important to examine the potential of this *in vitro* produced *R*. *irregularis* to increase cassava production in Nigerian savanna soils.

While attention was focused more on improvement of cassava yield through inoculation with efficient AMF species, one of the prime factors important for AMF inoculants to be effective is the carrier substrate selected for its mass multiplication and production. As a result, a group of researchers tried different carrier substrates with nutrient solutions. According to Azcon-Aguilar and Barea [25], a careful selection of functionally compatible host/fungus/substrate combination is critical for success. For this reason, we hypothesized that carrier substrates of inoculants used in this study could have influence on the cassava yield. On the other hand, high availability of P could greatly affect the response of cassava to AMF inoculation. While some reports showed that high soil P levels reduce AMF benefit by decreasing their association with plants (e.g. [26]), others indicated that high soil P does not always have a negative effect on AMF [27]. According to Howeler [28], inoculation of cassava with AMF increased plant growth and dry matter production only when P was applied. To this end, there is need for proper diagnosis of soil available P status and judicious combination of P and AMF inoculants in low fertility soils. This is particularly important in regions like sub-Saharan Africa where crop inoculation with AMF inoculants is not common. In this study we, therefore, assessed the potential of combining P fertilization and application of *in vitro*-produced AMF inoculants to improve cassava productivity in soils of the two regions: Northern Guinea savanna and Sudan savanna of Nigeria. In addition, we tested the effect of inoculant carriers of cassava productivity in the two regions.

## Materials and methods

### Experimental site description

The experiments were conducted at two sites of the Institute for Agricultural Research (IAR) stations i.e. Samaru and Minjibir. Samaru station is located in the Northern Guinea Savanna of Nigeria at N11°10´31.3´´, E007˚36´38.9´ and 704 m above sea level (masl). The soil of the experimental site belongs to an Alfisol classified as Typic Haplustalf overlying basement complex rocks [29]. The rainy season in Samaru is mono modal with average annual rainfall of 1011 ± 161 mm. The season starts in April and lasts until October, while the dry season is between November and March with mean maximum and minimum temperatures of 32.2°C and 18.9°C respectively. The site had been fallowed for several years and was covered with a variety of weeds and shrubs that are predominantly black doka (*Isoberlinia doka*). Minjibir station is located in the Sudan Savanna of Nigeria at N12° 08´31. 5´´, E008°40’15.5´´ and 436 masl. The soil belongs to an Alfisol classified as Aeric Halaquept [30]. The rainy season in Minjibir is also mono modal with average annual rainfall ranging between 800 mm and 900 mm. The site has been under cultivation and the previous crop was cowpea (*Vigna unguiculata*) which was removed after cultivation.

### Soil sampling and analysis

Soil samples for pre-planting analyses were taken at three depths: 0-15cm, 15-30cm and 30-45cm. Soil from the field was sampled by randomly selecting points in the field from which soil cores were taken. About 15 to 20 points were considered and soil cores were taken and bulked to make a pooled sample. The pooled soil sample was air dried, crushed and sieved through 2 mm mesh and prepared for laboratory analysis. Also, two profile pits were dug at each site and samples taken from each horizon for field characterization. The physical and chemical analyses were determined using standard methods thus: pH by the potentiometric method as described by [31]; organic carbon by chromic acid digestion [24]; cation exchange capacity (CEC) by saturation with 1 N NH_4_OAC and extraction of NH_4_OAC with 2 M KCl [32]; exchangeable acidity by the titration method after extraction with 1 N KCl [32]; ECEC by summation of exchangeable cations (Ca, Mg, K and Na) and exchangeable acidity; particle size analysis by the hydrometer method [33]; soil total nitrogen by digestion through wet oxidation based on Kjeldahl method [34]; and soil available P using Bray-1P [31]. These are reported in S1 Table. Also, approximate soil chemical characteristics based on nutritional requirement of cassava is presented in S2 Table.

### Field preparation and layout

The fields were cleared, ploughed, harrowed and ridged, with 75cm spacing, using a tractor to create a fine tilth. The fields were then divided according to the experimental treatments using pegs. There were 160 plots in total (i.e. 2 site, 20 treatments replicated 4 times) and each plot size was 9 m x 8 m. Planting was carried out on 1^st^ August 2014, during the rainy season. The cassava variety planted was TME 419; a high yielding variety released by the International Institute of Tropical Agriculture (IITA) and recommended for both regions. Cassava stem cuttings, approximately 25 cm long, were planted at a 1m x 0.75 m spacing giving a planting density of 13,333 plants per ha. The experiment was arranged in split plot design with phosphorus fertilizer rates as main plot and AMF inoculants as subplot. Treatments included four phosphorus fertilization levels (0, 17.5, 35 and 52.5 kg P_2_O_5_ ha^-1^) and five levels representing AMF inoculants or their carrier minus the fungus in the subplots (Glomygel, Glomygel carrier, Mycodrip, Mycodrip Carrier, and Control) in a full factorial experiment.

### Treatment application

The application of fertilizer was based on the recommendation made by the National Root Crop Research Institute (NCRI) of Nigeria, where optimal levels are considered as 90 kg N ha^-1^, 35 kg P_2_O_5_ ha^-1^, and 75 kg K_2_O ha^-1^. Sources for the fertilizers applied during the trials were urea (46% N), Single Super Phosphate (18% P_2_O_5_) and Muriate of Potash (60% K_2_O). While N and K were applied uniformly as recommended, P was applied according to the treatments. The side dressing method for fertilizer application was adopted to ensure adequate fertilization, i.e. the fertilizers were put beside the plant at approximately 5 cm distance from the stem.

Two AMF inoculants, each containing a different isolates of the AMF *R*. *irregularis* were produced *in vitro*, and their carrier substrates were obtained in concentrated form (approximately 1000 spores per plant) and diluted before application. The product names of these inoculants were Glomygel (Mycovitro S.L., Spain) and Mycodrip (Symbiom s.r.o., Czech Republic). Glomygel and its carrier were diluted in water in the ratio inoculant or carrier: water = 1: 3 while the Mycodrip inoculants and its blend carrier were diluted with sterile sand in the ratio inoculant or carrier: sand = 1: 9 (Producer’s recommendations). Inoculation was done at 20 days after planting when the stem cuttings had developed fine roots. This was done by carefully lifting the cassava stem cuttings and applying the inoculants beneath the stem cuttings. Two milliliters of Glomygel suspension, or its carrier, were applied. Two grams of the Mycodrip inoculant, or its carrier, were applied per each cassava stake.

The infective propagule numbers in the experimental soils were determined before inoculation (result is available in S1 Table).

### Harvesting

Harvesting was carried out at exactly 12 months after planting when the plants had attained maturity. This was done by hand pulling. A net plot of 4 m x 3 m consisting of 12 plants was marked out in each plot and the plant samples were taken therein. Both fresh weights of the roots, stem and leaves were taken *in situ* using a field scale. The subsamples of the leaves from each treatment were taken and oven dried at 70°C until constant weight.

### Plant tissue analysis

The dried leaf samples were ground, sieved and analyzed for selected nutrient concentrations. The concentrations of total nitrogen and phosphorus in plant tissue were measured in a digest following the methods outlined in [35]. Potassium concentration was measured by complete oxidation of the samples using the Kjeldahl procedure followed by spectrophotometric analysis [35]. Micronutrient concentration (Mn, Cu, Zn and Fe) was measured on dried leaf samples using an atomic absorption spectrophotometer, after being digested as outlined in the procedure by [35].

### Percentage root length colonization

Fine root samples of cassava were collected from each replicate of each treatment at harvesting and were washed in running tap water and stored in glass vials containing 50% ethanol for later analysis. The roots were then rinsed with water, at least four times, to remove the ethanol. The percent mycorrhizal colonization in cassava roots was determined according to the method outlined by [36]. The percentage root length colonized by AMF (% RLC) was determined by scoring the presence or absence of AMF crossing a graticule axis. Fifty intersections per slide were observed, counted and recorded. The % RLC was calculated using the following formula (1)

$$\%\;RLC=\frac{Number\;of\;root\;pieces\;showing\;colonization}{Total\;number\;of\;root\;pieces\;observed}\;x\;100$$ (1)

### Mycorrhizal response ratio (MR)

The mycorrhizal response ratio (MR) was determined to evaluate the effects of AMF inoculation on harvestable root yield, total plant biomass and leaf nutrient P concentration, using Eqs 2–4, respectively. An MR value > 1 implies an effective inoculation response [6].

$${MR}_{root\;yield}=\frac{{Root\;Yield}_{inoculated}}{{Root\;Yield}_{control}}$$ (2)

$${MR}_{Biomass}=\frac{{Biomass}_{inoculated}}{{Biomass}_{control}}$$ (3)

$${MR}_{Pconcentration}=\frac{{Pconcentration\;}_{inoculated}}{{Pconcentration}_{control}}$$ (4)

### Statistical analysis

All data collected were fit into general linear model (GLM) and subjected to analysis of variance using SAS software version 9.4. Where significant F values were observed at a significance level of p < 0.05, a Tukey honest significant difference (HSD) test was used to separate the means. Also, Pearson correlation coefficient was used to examine the relationship between measured parameters. To compare the effects of two locations, the data from two locations were pooled and analyzed (S3 Table)

## Result

### Soil properties

The fertility trait of the soils was mostly similar except for higher organic matter in the Samaru than Minjibir and the higher available P in Minjibir (S1 Table). Plant available P was higher in Minjibir and was also higher than critical level of 7 mg P kg^-1^ soil (Bray 1 extract) for cassava production (S2 Table). The soils were moderately acidic, which was in agreement with the general characteristics of an Alfisol soil order of the tropics but within the suitable range for cassava production, as defined by [14]. The total N and organic carbon of both sites were very low (S1 Table). The exchangeable Ca was high in both soils while K fell within the medium classification for cassava production. Only Mg concentration was in the lowest class (S2 Table).

The physical characteristics of the soils showed great variability. The surface soil of Samaru was silt loam in nature and had a high percentage of silt and clay in the subsurface (S1 Table). This indicated that the soil is higher in pore spaces with lower pore volume space and poor drainage, having high clay content and, thus, shallow in nature. These characteristics are conducive to surface run off and waterlogging, and consequently, formation of a hardpan layer, that would restrict cassava root formation. The soil of Minjibir was loamy sand and had a good proportion of soil particles. The soil was deep, friable and well aerated which allows drainage. These attributes are favorable to good cassava root formation.

### Yield and growth of cassava

**Fig 1:**
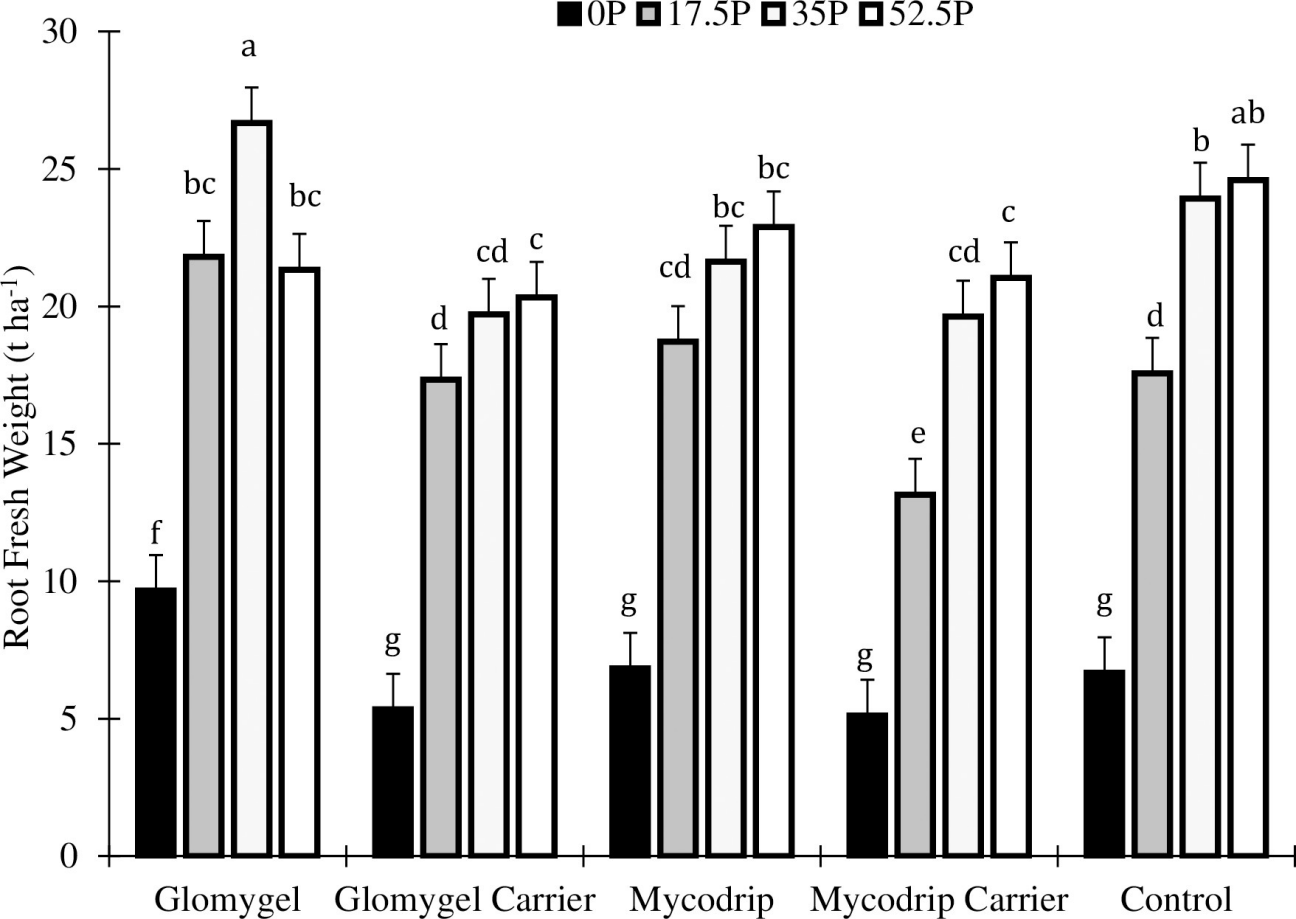
Effects of different levels of P fertilizer application and inoculation with AMF on cassava root fresh weight in Samaru.

Analysis of the root fresh weight of cassava in Samaru revealed a highly significant interaction between phosphorus (P) and Inoculants (I) on root fresh yield (Table 1). Root fresh yield per hectare was affected by both P fertilization and inoculation with AMF at Samaru (Fig 1). However, the effect of AMF inoculation was not the same in all P fertilization treatments as shown by a significant P x I interaction. For example, inoculation with Glomygel increased cassava yield at all P levels except 52.5 kg P_2_O_5_ ha^-1^ compared to the control and the carrier. At 0 kg P_2_O_5_ ha^-1^, inoculation with Glomygel significantly increased cassava fresh yield over inoculation with all the other inoculants including the control (Fig 1). Here, inoculation with Glomygel increased cassava fresh yield by 81%, 41%, 89% and 45% over the Glomygel carrier, Mycodrip, Mycodrip carrier and the control respectively. Mycodrip carrier appeared to have a negative effect on plant growth compared to Mycodrip or the control.

**Table 1:** Summary table indicating the main effects of P fertilizers and AMF inoculants on the measured parameter F–values (p–values).

	Samaru			Minjibir		
Parameters	Phosphorus (P)	Inoculants (I)	Interaction P x I	Phosphorus (P)	Inoculants (I)	Interaction P x I
Root	315.00 (<0.0001)	19.66 (<0.0001)	3.28 (0.0016)	0.36 (0.78)	0.13 (0.97)	1.51 (0.15)
Shoot	32.67 (<0.0010)	5.82 (0.0007)	1.20 (0.3119)	1.51 (0.22)	0.54 (0.71)	1.86 (0.06)
Root/Shoot	136.46 (<0.0001)	3.22 (0.0202)	2.07 (0.0382)	0.83 (0.48)	0.59 (0.67)	1.57 (0.13)
Total Plant	195.86 (<0.0001)	16.07 (<0.0001)	2.35 (0.0182)	0.56 (0.64)	0.24 (0.92)	1.63 (0.11)
N concentration	74.87 (<0.0001)	5.90 (0.0006)	1.12 (0.3698)	0.61 (0.63)	0.13 (0.97)	0.49 (0.91)
P concentration	128.75 (<0.0001)	3.25 (0.0195)	1.56 (0.1354)	0.40 (0.75)	0.28 (0.89)	0.53 (0.88)
K concentration	6.40 (<0.0001)	2.65 (0.0447)	1.56 (0.1368)	0.66 (0.60)	0.52 (0.72)	0.59 (0.84)
Zn concentration	51.27 (<0.0001)	4.93 (0.0021)	0.78 (0.6714)	3.54 (0.0214)	1.11 (0.36)	0.81 (0.64)
Mn concentration	80.47 (<0.0001)	2.05 (0.102)	1.15 (0.3473)	1.02 (0.39)	1.21 (0.32)	0.62 (0.81)
Cu concentration	15.63 (<0.0001)	2.17 (0.087)	1.32 (0.2368)	2.16 (0.11)	1.97 (0.11)	1.20 (0.31)
Fe concentration	48.61 (<0.0001)	2.05 (0.1026)	1.61 (0.1206)	3.96 (0.0133)	0.35 (0.84)	1.36 (0.22)
PRC	10.10 (<0.0001)	5.87 (0.0006)	1.04 (0.43)	0.44 (0.72)	0.24 (0.91)	0.08 (1.00)
MR root	2.88 (0.0457)	14.49 (<0.0001)	2.62 (0.0089)	0.25 (0.86)	0.21 (0.93)	1.60 (0.13)
MR biomass	5.62 (0.0022)	11.64 (<0.0001)	1.67 (0.10)	0.65 (0.59)	0.25 (0.91)	1.56 (0.14)
MR P concentration	11.4 (<0.0001)	0.85 (0.4998)	1.70 (0.097)	0.16 (0.93)	0.13 (0.97)	0.02 (1.00)

Sources of variation: P, I, and P × I.; for each measured parameter, the values in front of the brackets are F test-values, while the values between the brackets are p-values

**Fig 2:**
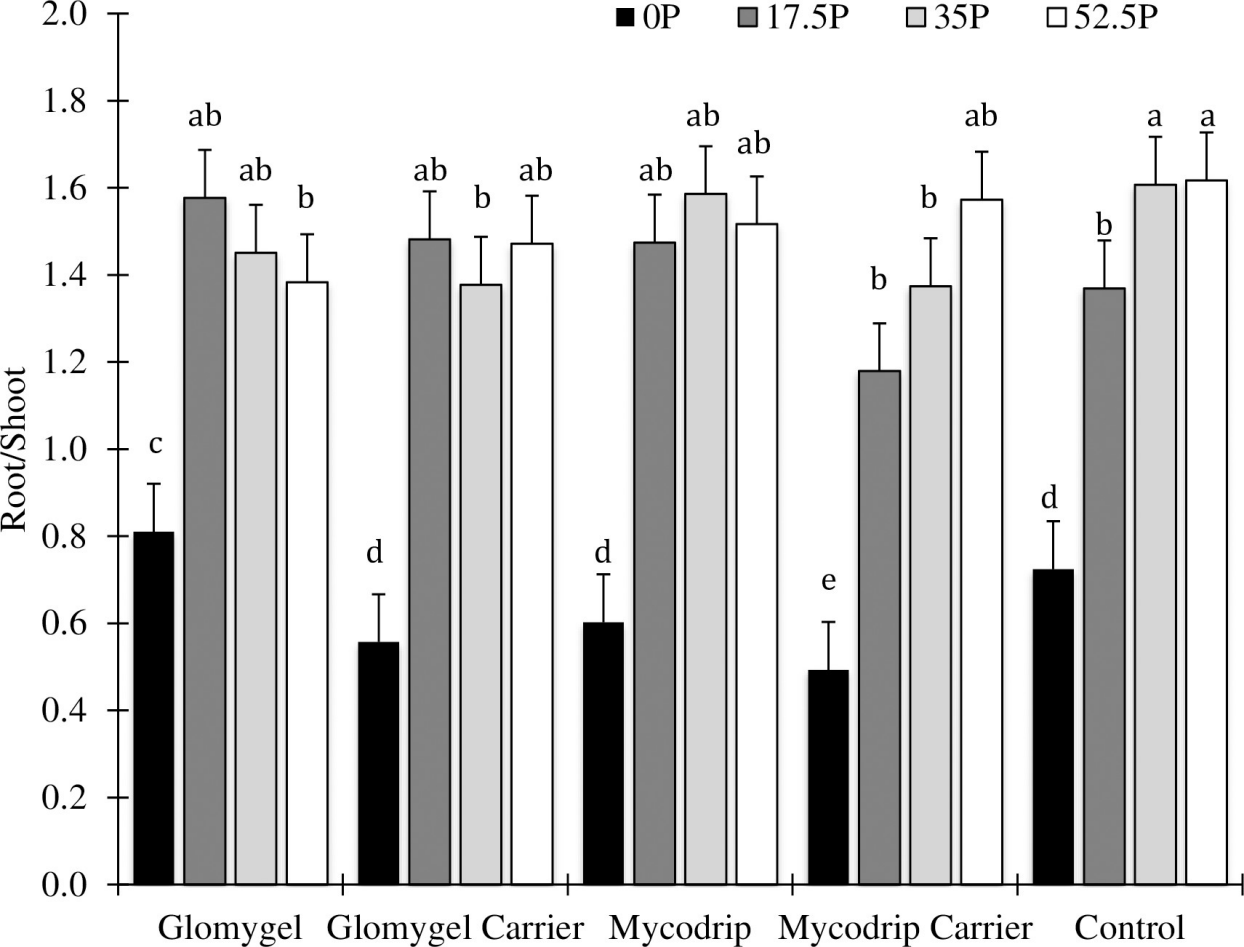
Effects of different levels of P fertilizer application and inoculation with AMF on cassava root: Shoot ratio in Samaru.

Significant differences in root/shoot ratio were also observed as a result of P fertilization and AMF inoculation in Samaru (Table 1). The highest ratio was obtained when 52.5 kg P_2_O_5_ ha^-1^ was applied (Fig 2). The combinations of 17.5 kg P_2_O_5_ ha^-1^ + Glomygel and 17.5 kg P_2_O_5_ ha^-1^ + Mycodrip were statistically similar in apportioning of biomass compared to when 52.5 kg P_2_O_5_ ha^-1^ was applied (Fig 2). Inoculation with Glomygel at 0 kg P_2_O_5_ ha^-1^ significantly increased the root/shoot ratio over other treatments. Here also the inoculation with Mycodrip carrier recorded lowest yield.

**Fig 3:**
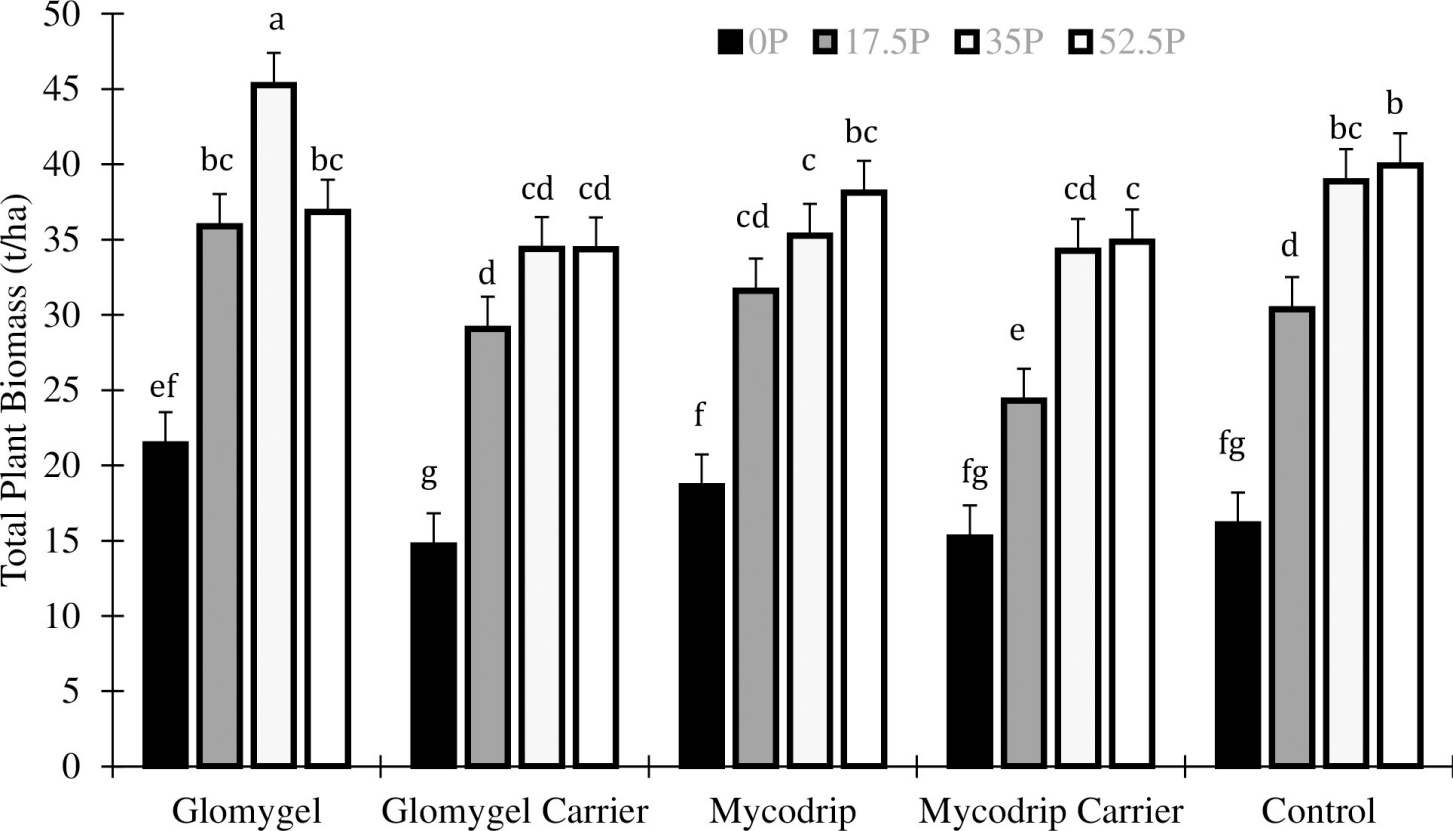
Effects of different levels of P fertilizer application and inoculation with AMF on total cassava biomass in Samaru.

There was an interaction between P and I on total plant biomass which showed that highest total plant biomass yield was obtained with the application of 35 kg P_2_O_5_ ha^-1^ + Glomygel (as in root fresh yield) and this was statistically higher than all other treatments. Application of 17.5 kg P_2_O_5_ ha^-1^ + Glomygel gave a total plant biomass that was statistically similar to 35 kg P_2_O_5_ ha^-1^ (Fig 3). Additionally, the application of 17.5 kg P_2_O_5_ ha^-1^ + Glomygel increased the total plant biomass significantly over application of 17.5 kg P_2_O_5_ ha^-1^ in the control (Fig 3).

Analysis of main and sub effects and the interaction of the treatments in Minjibir were not significant in all the yield components of cassava.

### Nutrient concentration

**Fig 4:**
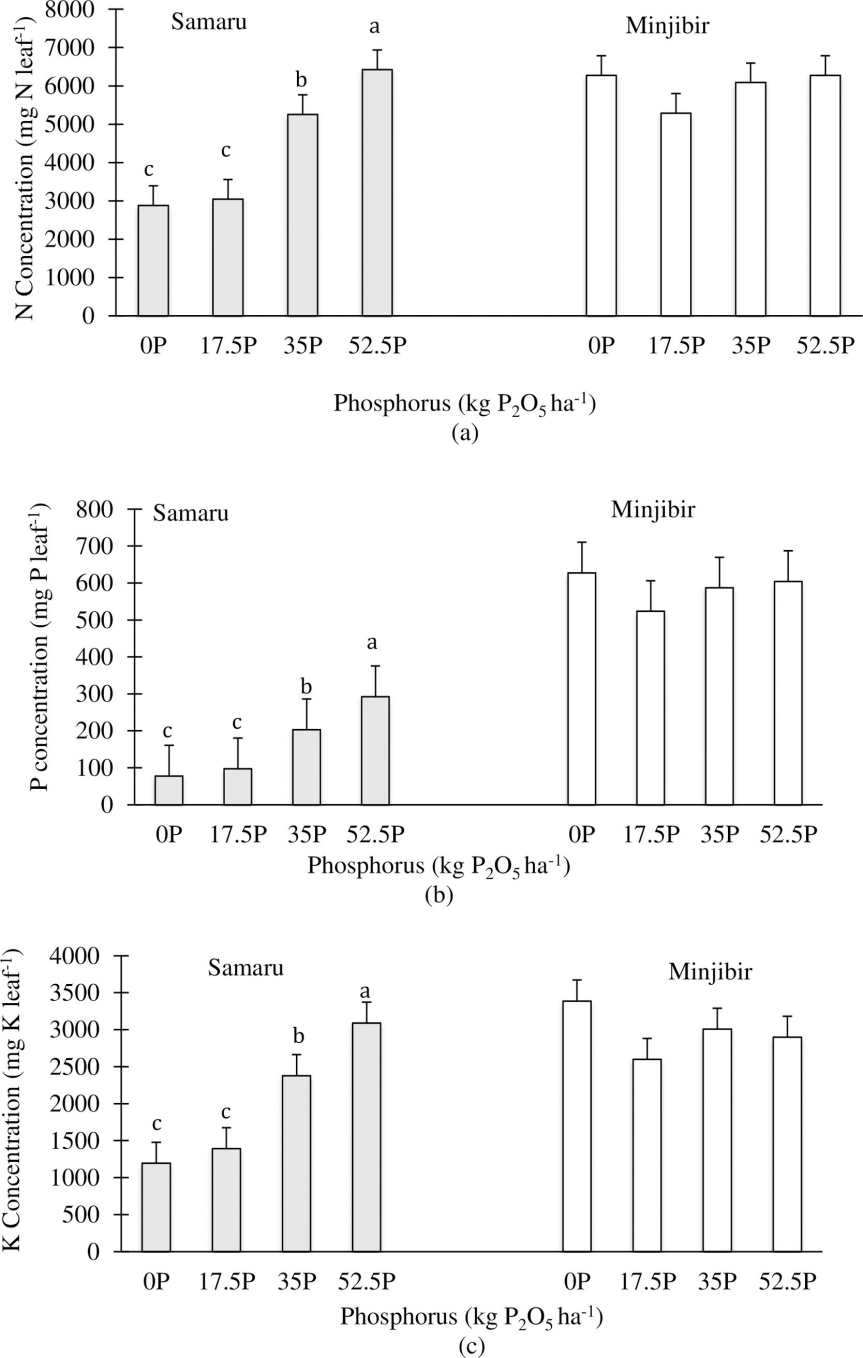
Main P treatment effects on leaf macronutrient concentrations in cassava. (a) Mean leaf N concentration (b) Mean leaf P concentration and (c) Mean leaf K concentration at both trial sites (Samaru and Minjibir) averaged over all the inoculation levels for the four different P fertilizer levels.

There were no significant P x I interactions in terms of leaf macronutrient concentrations at both sites (Table 1). However, in Samaru, analysis of the main and sub effects showed variation in the leaf macronutrient concentrations (Table 1). Application of P increased the leaf macronutrient concentrations proportional to P application i.e. an increase in P application improved the macronutrient concentration (Fig 4A, Fig 4B, Fig 4C). Increasing P application resulted in increased N leaf concentrations by 6%, 83% and 123% (Fig 4A); P concentration by 25%, 161% and 273% (Fig 4B); and K concentration by 17%, 99% and 159% (Fig 4C) for 17.5 kg P_2_O_5_ ha^-1^, 35 kg P_2_O_5_ ha^-1^ and 52.5 kg P_2_O_5_ ha^-1^ respectively.

**Fig 5:**
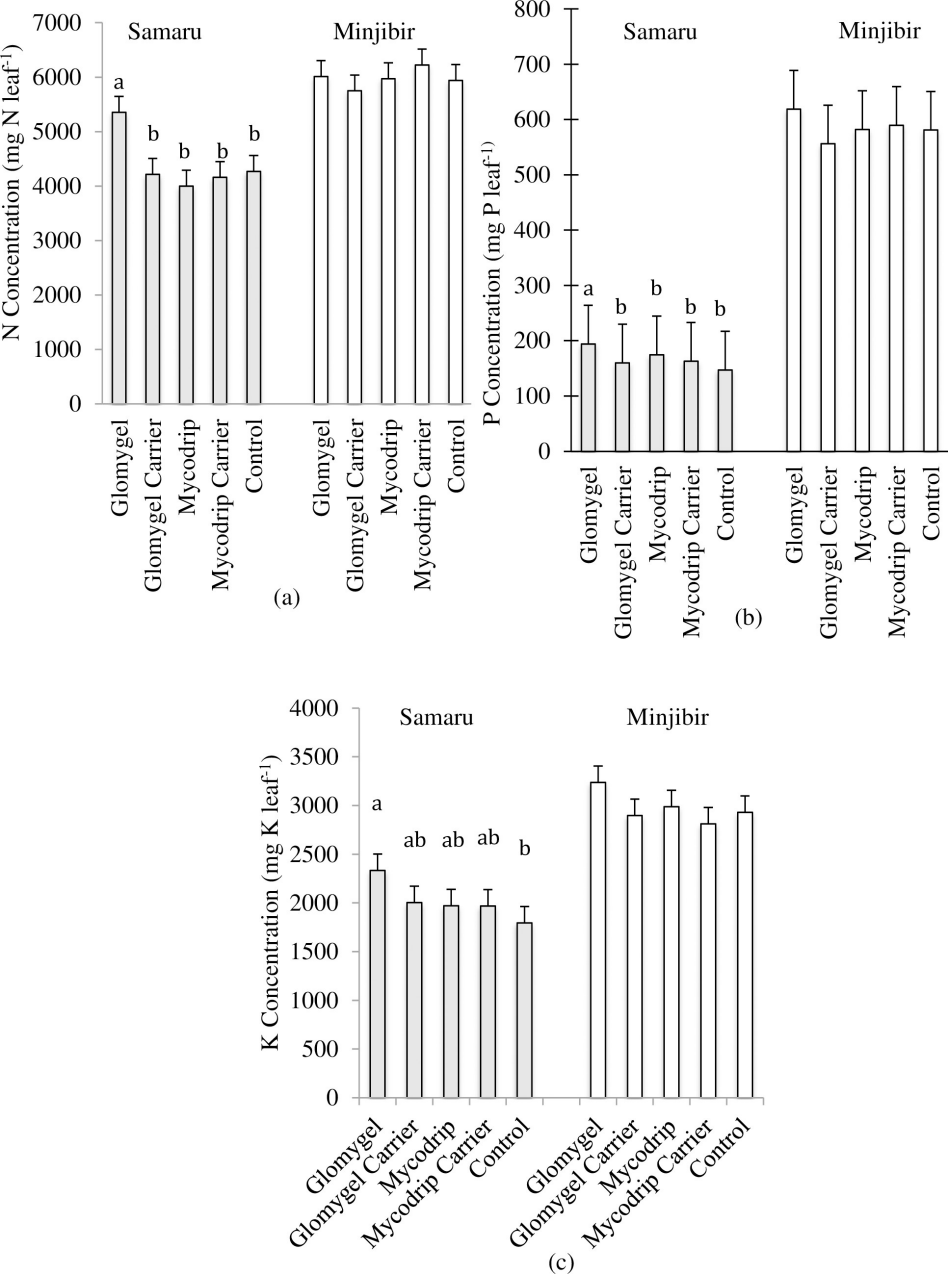
Main inoculation treatment effects on leaf macronutrient concentrations in cassava. (a) Mean leaf N concentration (b) Mean leaf P concentration and (c) Mean leaf K concentration at both trial sites ((Samaru and Minjibir) averaged over all P treatment- levels for the five inoculation levels.

Leaf macronutrient concentrations also differed significantly among cassava treated with different inoculants (Table 1). Cassava inoculated with Glomygel resulted in significantly higher N concentration compared with other treatments. The magnitude of difference in N concentration between cassava inoculated with Glomygel and other treatments was 27% more than the Glomygel carrier, 34% more than Mycodrip, 29% more than the Mycodrip carrier and 25% more than control (Fig 5A). A similar trend was observed in both P and K concentrations although Glomygel was not statistically different from Mycodrip (Fig 5B and Fig 5C). Application of P at 52.5 kg P_2_O_5_ ha^-1^ resulted in significantly higher macronutrient leaf concentration when compared with application of 35 kg P_2_O_5_ ha^-1^ and 17.5 kg P_2_O_5_ ha^-1^. Also, application of 35 kg P_2_O_5_ ha^-1^ resulted in significantly different micronutrient concentrations to plants with 17.5 kg P_2_O_5_ ha^-1^; but there was no difference between application of 17.5 kg P_2_O_5_ ha^-1^ and 0 kg P_2_O_5_ ha^-1^. On the other hand, there was a significantly different leaf N and K concentrations of cassava inoculated with Glomygel compared to plants inoculated with Mycodrip. There were no significant effects on the leaf macronutrient concentrations as a result of P applications and AMF inoculation in Minjibir (Table 1).

There were no significant P x I interactions in micronutrient concentration at both locations (Table 1). However, P application increased micronutrient concentration at Samaru. Significant variation was also found in the leaf micronutrient concentrations as a result of AMF inoculation. Inoculation with Glomygel resulted in a significantly higher Zn concentrations compared to inoculation with Mycodrip and the carrier materials (Table 1). In Minjibir, analysis of Zn and Fe concentrations recorded a significant difference between the levels of P where application at 17.5 kg P_2_O_5_ ha^-1^ recorded lower concentration compared to other treatments (Table 1).

### Percentage root colonization (PRC)

**Fig 6:**
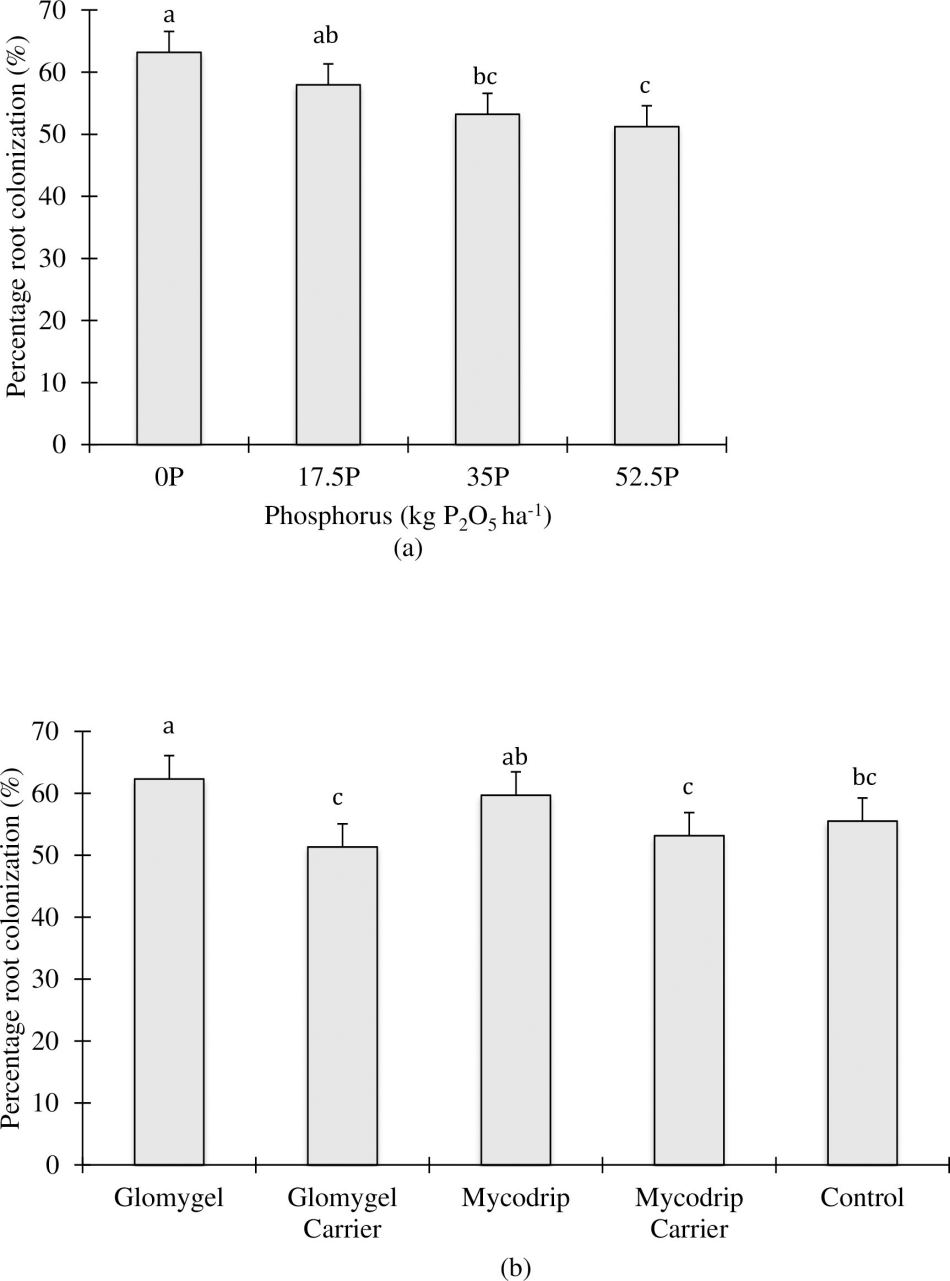
Percentage of root length colonized by AMF in cassava at the Samaru trial site. (a) Main P treatment effect; (b) main inoculation treatment effect.

Analysis of PRC showed that there was no significant P x I interaction in both locations. However, in Samaru, main- and sub- treatment effects showed that there were variations among the treatments (Table 1; Fig 6A). There were also variations in PRC as a result of AMF inoculations (Table1; Fig 6B). Inoculation with Glomygel recorded a significantly higher PRC over its carrier and control while inoculation with Mycodrip recorded a significantly higher PRC than its carrier though not statistically different from control (Fig 6B). There was no significant PRC recorded in Minjibir at both main and sub treatment effects (Table 1).

### Mycorrhizal response (MR) ratio

**Fig 7:**
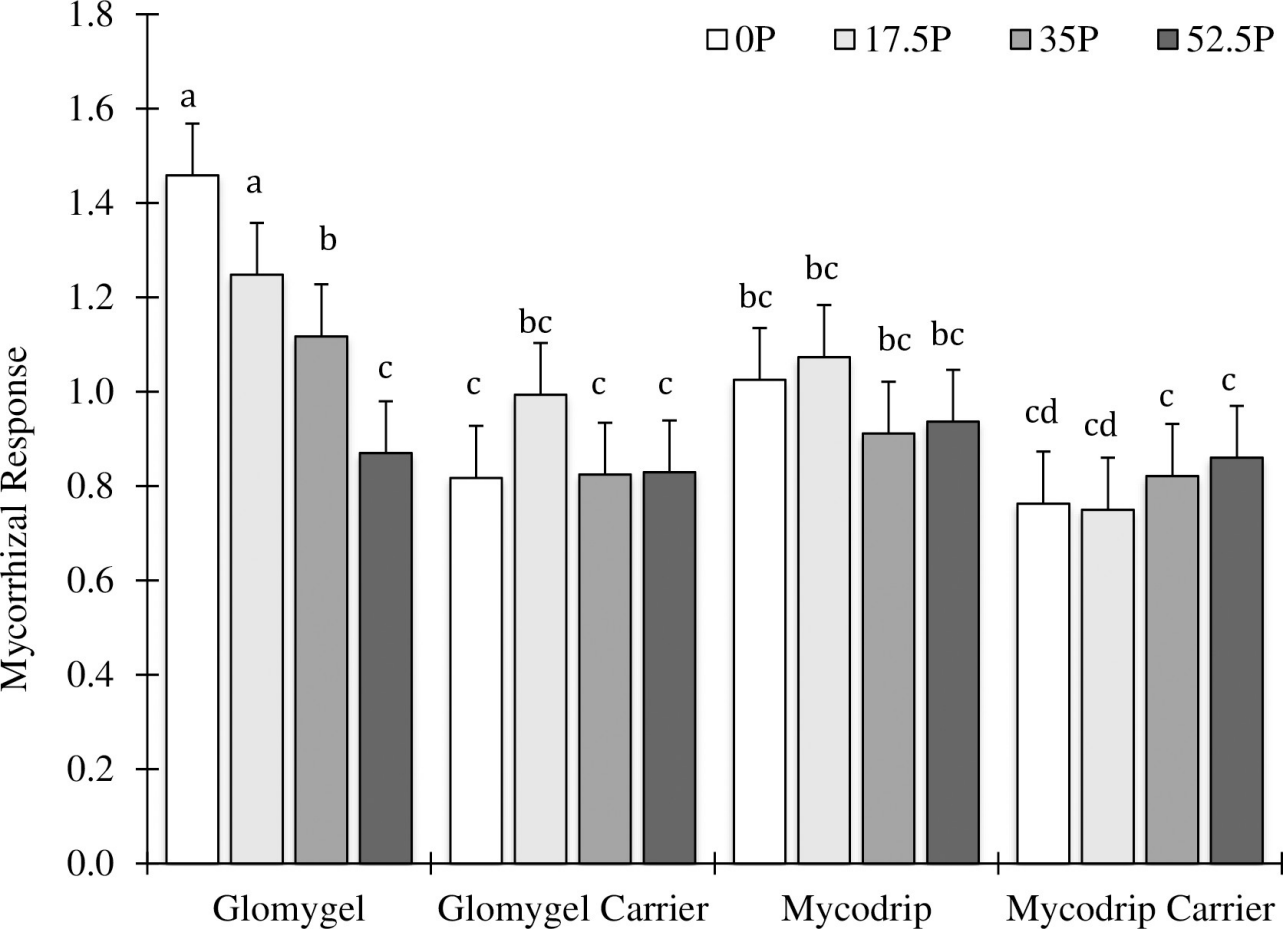
Mean mycorrhizal response of cassava for the different inoculation levels at Samaru across the different P levels.

Analysis of MR of root fresh yield in Samaru showed that there was significant P x I interaction (Table 1; Fig 7). Here, application of 0 kg P_2_O_5_ ha^-1^ + Glomygel recorded highest MR. Also, combination of 17.5 kg P_2_O_5_ ha^-1^ + Glomygel and 35 kg P_2_O_5_ ha^-1^ + Glomygel recorded MR that is above a unity. Similar trend was observed in Mycodrip in which the combination 17.5 Kg P_2_O_5_ ha^-1^ + Mycodrip was found to be greater than a unity (Fig 7). There were no P x I interactions in both biomass and P concentration in Samaru (Table 1). There were no P x I interactions recorded in MR ratios (root yield, biomass and P concentration) in Minjibir (Table 1). Analysis of the main and sub-treatment effects in Samaru however, showed that there was significant difference in MR in the total plant fresh biomass where application of 0 and 17.5 Kg P_2_O_5_ ha^-1^ showed higher MR when compared with application of 35 Kg P_2_O_5_ ha^-1^ and 52.5 Kg P_2_O_5_ ha^-1^. No main and sub effects were recorded in Minjibir for MR.

### Correlation between the measured parameters

Correlation analysis was carried out to relate the measured variables with one another (S4 Table). In Samaru, high correlations between yield components of cassava (root fresh weights, shoot fresh weights and root/shoot ratio) and leaf nutrient concentrations were highly significant (S4 Table). Moreover, PRC significantly correlated with the leaf nutrient concentrations showing higher nutrient uptakes with more PRC. Also, significant (*p*<0.05) correlation between PRC and MR root fresh yield (*r* = 0.216*) was recorded which shows there was increased harvestable root as a result of increased PRC.

## Discussion

### Site effect of co-applied P fertilizer and *in-vitro* AMF on cassava yield

The response of cassava to co-application of P and AMF inoculants was large in Samaru. However, in Minjibir there were almost no effects of P fertilization or AMF inoculation for almost all of the variables. Yield was much higher in Minjibir irrespective of treatment application, indicating that soil fertility at this site is much better than Samaru for cassava cultivation. The disparity in effects of the treatments between the two locations is likely to be the result of inherent differences in the properties of the soils such as texture, structure or compaction (S1 Table). The higher yield obtained in Minjibir was likely due to the fact that the soil was light textured, deep and better drained than Samaru. The soil was moderately suitable due to its medium level of soil chemical characteristics in comparison with the nutrient requirement for cassava production, as reported by [14]. The variability of the biological, physical and chemical properties of soil is a major reason for the variability of cassava production [37,38]. In comparison, the Samaru soil was less suitable as it was shallow in nature, having higher silt and clay contents (S1 Table) that would result in high susceptibility to compaction. Soil physical characteristics are likely to affect the development of cassava variety TME 419, which will produce tuberous roots to 1 m deep under favorable conditions such as those found at the site in Minjibir.

### Effect of co-applying P fertilizer and *in vitro* AMF inoculants on cassava yield

The application of P fertilizer and the AMF inoculants in Samaru showed that the maximum cassava yield could be obtained by applying 66% of the full recommended P fertilization and one of the *in vitro* AMF inoculants (Glomygel). This yield was statistically similar to the situation where full P fertilizer was applied with no AMF inoculant (Fig 1). This shows that the recommended P fertilizer rate can be reduced (representing 33.3% P fertilizer reduction) in combination with one of the *in vitro* AMF inoculants in some soils. This is an important result, which indicates that farmers in the region can potentially use these inoculants to reduce P fertilizer usage. This finding is in line with a report by [23] who found that *in vitro* produced AMF could be used as an addition in order to reduce P fertilizer application.

The results showed that cassava yield in Samaru was increased by Glomygel at all levels of P fertilization, except at the recommended dose of P. Cassava that was not inoculated actually had a similar yield at the full recommended level of P application compared to the 35 kg P_2_O_5_ ha^-1^ level. This indicates that at this location, 66.6% of the recommended application of P fertilizer represents a more realistic optimum for growing cassava and that within this range of P fertilization, the AMF inoculant Glomygel, enhanced yields. This means that if the farmer inoculates the crop with AMF but does not apply any P fertilizer, or only a small amount of P fertilizer, then it is possible to get a significant growth response.

The mycorrhizal responsiveness of cassava to inoculation with the fungus differed according to the P fertilization treatment. When cassava was inoculated with Glomygel, the mycorrhizal responsiveness (essentially a measure of the efficacy of inoculation) was proportionally greater at no or low P fertilization than at higher P fertilization levels (Fig 7), even though overall yield of cassava at low P application was lower than at high P application. This would be expected given that there are many reports in literature that higher soil P levels reduce the mycorrhizal benefit by decreasing the AMF association with plants [26, 39, 40]. It was reported by [41] that formation and growth of AMF might only be negatively affected at high P supply levels when P is applied homogenously to the soil in pot experiments under controlled conditions, but this experiment indicates that such an effect is observable in field conditions. In this experiment, indeed, colonization of the cassava roots by AMF decreased with increasing P application in field conditions.

The two inoculants both contained the same species of fungus, but were different strains. The Mycodrip inoculant gave significantly higher cassava yields at some P fertilization levels than the Mycodrip carrier. However, the experiments showed a clear negative effect of the carrier alone (without AMF) on the yield of cassava. Thus, it may be that the fungal strain in Mycodrip also positively affects cassava growth but that the effect of the fungus was masked by the negative effect of the carrier.

It is impossible in these experiments to know the mechanism by which inoculation with Glomygel lead to an increase in cassava yield under the field conditions at Samaru. Irrespective of inoculation, cassava is naturally colonized by the local community of AMF. Thus, the effects of inoculation could either be due to a direct effect of the inoculated fungal strain on plant growth and P acquisition or through an indirect effect of the inoculated strain on the local AMF community or the other components of the soil microbiome [16]. Inoculation with Glomygel led to significantly higher levels of AMF colonization in cassava roots compared to the control or the carrier alone but we cannot say whether this was an increase in the colonization of cassava roots by the inoculant fungus or other members of the local AMF community. At present, molecular markers do not exist to track specific AMF individuals of this fungus in the roots, although with the generation of population genomic data on genetic variation in this fungal species this may be possible in the future [42].

### Effects of co-applying P fertilizer and *in vitro* AMF inoculants on cassava nutrient concentration

One notable feature of the results was that cassava root yield and overall biomass accumulation at Samaru was affected by both P application and *in vitro* AMF inoculants, but that these two factors interacted with each other. Nutrient concentration was also affected by these two factors but strikingly these two factors did not interact. Increasing P application resulted in the concentration of increasingly more nutrients irrespective of the inoculation treatments. Inoculation with AMF only resulted in a significantly higher uptake of nutrients in the treatment with Glomygel, irrespective of P treatment.

## Conclusion

Co-application of P fertilizer and *in vitro* AMF inoculants in the Northern Guinea Savanna (Samaru) and Sudan Savanna (Minjibir) of Nigeria showed a strong site effect demonstrating the need of local adaptation of the practices. Such a strong site effect also called for further investigation to identify AMF strains that could either perform at scale or specific to various locations. In the Samaru site, co-application of P and *in vitro* produced AMF led to improved yield components of cassava and at 66% of the current recommended P rate for cassava in the region in the presence of Glomygel. The yield increase offered by Glomygel at Samaru with the same level of P fertilization was up to 12% yield increase, which represents a significant yield increase for the farmer. Given that the inoculant is very easy to apply at planting, inoculation requires little extra labor cost for the farmer or special management throughout the crop cycle. Validation of this finding across the Northern Guinea Savanna of Nigeria and beyond and local availability of AMF inoculants would be important, as it would represent a significant cost reduction on P fertilizer for the resource-constrained farmers who represent the majority of the farming community in the region. This finding is crucial to establish potential local formulation of AMF inoculants using such strains, which could improve local availability of the products. It would be important to assess the reproducibility of the findings at a larger scale and in time using for instance various AMF strains to better inform the incorporation of effective AMF inoculants in integrated soil fertility management to improve cassava production in sub-Saharan Africa in general, and in Nigeria in particular.

## Supporting information

S1 Fig Experimental lay-out at both sites i.e. Samaru and Minjibir. A. Phosphurus levels: P0 = 0 kg P_2_O_5_ ha^-1^; P50 = 17.5 kg P_2_O_5_ ha^-1^; P100 = 35 kg P_2_O_5_ ha^-1^; P150 = 52.5 kg P_2_O_5_ ha^-1^. B. Arbuscular Mycorrhical Fungi levels: AMF + 1 = Glomygel Inoculant; AMF– 1 = Glomygel Carrier; AMF + 2 = Mycodrip Inoculants; AMF– 2 = Mycodrip Carrier; Control = absence of inoculant and carrier.
(TIF)

S1 Table Physical and chemical analyses and AMF count of the experimental sites ± standard deviation. The infective propagule was determined at soil depth of 0–5 cm.
(DOCX)

S2 Table Approximate classification for soil chemical characteristics according to the nutritional requirement of cassava. Adapted from [43]
(DOCX)

S3 Table Effect of location on measured parameters when data from the two sites (i.e. Samaru and Minjibir) were pooled. (DOCX)

S4 Table Correlation between nutrient concentrations and yield component of cassava. (DOCX)
