# Supplementary figures and images for: Effect of co-application of phosphorus fertilizer and in vitro-produced mycorrhizal fungal inoculants on yield and leaf nutrient concentration of cassava
Source: PLoS One. 2019 Jun 26;14(6):e0218969. doi: 10.1371/journal.pone.0218969 (PMC6594633; doi:10.1371/journal.pone.0218969)

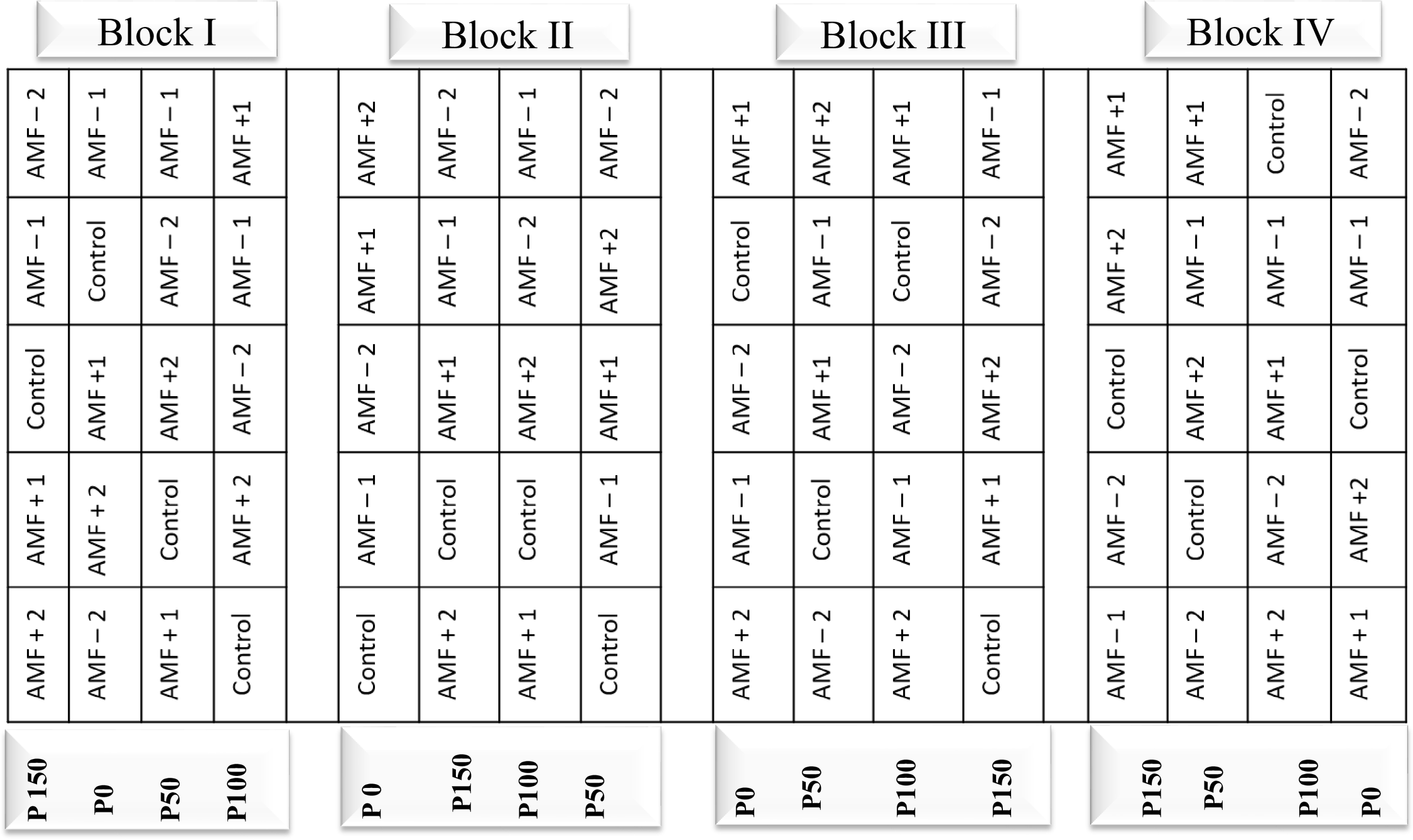

Supplement: S1 Fig — A. Phosphurus levels: P0 = 0 kg P2O5 ha-1; P50 = 17.5 kg P2O5 ha-1; P100 = 35 kg P2O5 ha-1; P150 = 52.5 kg P2O5 ha-1. B. Arbuscular Mycorrhical Fungi levels: AMF + 1 = Glomygel Inoculant; AMF– 1 = Glomygel Carrier; AMF + 2 = Mycodrip Inoculants; AMF– 2 = Mycodrip Carrier; Control = absence of inoculant and carrier. (TIF) [file pone.0218969.s001.tif]
