# Supplementary material for: Effect of co-application of phosphorus fertilizer and in vitro-produced mycorrhizal fungal inoculants on yield and leaf nutrient concentration of cassava
Source: PLoS One. 2019 Jun 26;14(6):e0218969. doi: 10.1371/journal.pone.0218969 (PMC6594633; doi:10.1371/journal.pone.0218969)
Supplement: S1 Table — The infective propagule was determined at soil depth of 0–5 cm. (DOCX) [file pone.0218969.s002.docx]

| Property | | Samaru | | | Minjibir | | |  |
| --- | --- | --- | --- | --- | --- | --- | --- | --- |
|  |  | Depth (cm) | | | Depth (cm) | | |  |
|  |  | 0-15 | 15-30 | 30-45 | 0-15 | 15-30 | 30-45 | |
| Sand (%) |  | 37 ± 1.41 | 31 ± 1.41 | 33 ± 1.41 | 82 ± 0.00 | 82 ± 2.83 | 79 ± 1.41 | |
| Silt (%) |  | 51 ± 1.41 | 50 ± 0.00 | 43 ± 1.41 | 10 ± 0.00 | 10 ± 2.83 | 11 ± 1.41 | |
| Clay (%) |  | 12 ± 0.00 | 19 ± 1.41 | 24 ± 0.00 | 8 ± 0.00 | 8 ± 0.00 | 10 ± 0.00 | |
| Textural Class |  | Silt Loam | Silt Loam | Loam | Loamy Sand | Loamy Sand | Sandy Loam | |
| pH (H_2_O) |  | 6.10 ± 0.01 | 5.76 ± 0.01 | 6.72 ± 1.41 | 6.27 ± 0.08 | 6.34 ± 0.01 | 6.41 ± 0.00 | |
| Total N (gkg^-1^) |  | 0.70 ± 0.00 | 0.70 ± 0.50 | 1.05 ± 0.00 | 0.88 ± 0.25 | 0.88 ± 0.25 | 1.05 ± 0.50 | |
| Organic C (gkg^-1^) |  | 6.25 ± 1.49 | 5.00 ± 1.32 | 3.95 ± 1.20 | 3.55 ± 0.35 | 3.15 ± 0.64 | 3.20 ± 0.14 | |
| Available P (mgkg^-1^) |  | 1.89 ± 0.00 | 1.14 ± 0.01 | 1.37 ± 0.00 | 10.92 ± 0.50 | 9.62 ± 0.27 | 4.50 ± 0.54 | |
| Exch. Bases (Cmol^(+)^ kg^-1^)  Ca |  | 5.92 ± 0.10 | 6.04 ± 0.57 | 6.13 ± 0.18 | 5.06 ± 0.57 | 6.61 ± 0.16 | 5.45 ± 1.17 | |
| Mg |  | 0.44 ± 0.05 | 0.44 ± 0.05 | 0.43 ± 0.00 | 0.34 ± 0.01 | 0.41 ± 0.01 | 0.34 ± 0.06 | |
| K |  | 0.16 ± 0.10 | 0.14 ± 0.01 | 0.17 ± 0.00 | 0.14 ± 0.00 | 0.10 ± 0.01 | 0.09 ± 0.00 | |
| Na |  | 0.55 ± 0.09 | 0.48 ± 0.01 | 0.56 ± 0.01 | 0.56 ± 0.50 | 0.60 ± 0.04 | 0.51 ± 0.16 | |
| Exch. Acidity (Cmol^(+)^ kg^-1^) |  | 0.04 ± 0.00 | 0.60 ± 0.00 | 0.60 ± 0.00 | 0.04 ± 0.00 | 0.60 ± 0.00 | 0.04 ± 0.00 | |
| ECEC (Cmol^(+)^ kg^-1^) |  | 7.47 ± 0.05 | 7.75 ± 0.64 | 7.92 ± 0.16 | 6.53 ± 0.53 | 8.32 ± 0.15 | 7.00 ± 1.13 | |
| AMF Infective propagules (g^-1^ soil) |  | 3 |  |  | 2 |  |  | |

S1 Table
