# Supplementary material for: Effect of co-application of phosphorus fertilizer and in vitro-produced mycorrhizal fungal inoculants on yield and leaf nutrient concentration of cassava
Source: PLoS One. 2019 Jun 26;14(6):e0218969. doi: 10.1371/journal.pone.0218969 (PMC6594633; doi:10.1371/journal.pone.0218969)
Supplement: S2 Table — Adapted from [43] (DOCX) [file pone.0218969.s003.docx]

| Soil Parameter | Very low | Low | Medium | High | Very high |
| --- | --- | --- | --- | --- | --- |
| pH | <3.5 | 3.5-4.5 | 4.5-7.0 | 7.0-8.0 | >8.0 |
| Organic matter (gkg^-1^) | <1 | 1.0-2.0 | 2.0-4.0 | >4.0 | - |
| P (mgkg^-1^) | <2 | 2.0- 4.0 | 10.0-14.0 | >14.0 | - |
| K (Cmol^(+)^ kg^-1^) | - | <1.0 | 0.1- 0.15 | 0.15 -0.25 | >0.25 |
| Ca (Cmol^(+)^ kg^-1^) | <0.25 | 0.25-1.00 | 1.0 - 5.0 | >5.0 | - |
| Mg (Cmol^(+)^ kg^-1^) | <0.2 | 0.2 - 0.4 | 0.4 - 1.0 | >1.0 | - |

S2 Table
