# Supplementary material for: Effect of co-application of phosphorus fertilizer and in vitro-produced mycorrhizal fungal inoculants on yield and leaf nutrient concentration of cassava
Source: PLoS One. 2019 Jun 26;14(6):e0218969. doi: 10.1371/journal.pone.0218969 (PMC6594633; doi:10.1371/journal.pone.0218969)
Supplement: S3 Table — (DOCX) [file pone.0218969.s004.docx]

S3 Table

| Parameters | Location | | F- value (p-value) |
| --- | --- | --- | --- |
|  | Samaru | Minjibir |  |
| Root (tons/ha) | 17.19b | 32.39a | 205.05 (<.0001) |
| Shoot (tons/ha) | 13.27b | 38.94a | 287.01 (<.0001) |
| Root/Shoot | 1.26a | 0.89b | 25.31 (<.0001) |
| Total Plant (tons/ha) | 30.46b | 71.33a | 295.26 (<.0001) |
| N Concentration (mg N/leaf) | 4400.93b | 5980.47a | 49.26 (<.0001) |
| P Concentration (mg P/leaf) | 167.76b | 585.54a | 335.69 (<.0001) |
| K Concentration (mg K/leaf) | 2013.57b | 2972.56a | 50.74 (<.0001) |
| Zn Concentration (mg Zn/leaf) | 24.95b | 31.16a | 19.52 (<.0001) |
| Mn Concentration (mg Mn/leaf) | 24.78b | 31.70a | 23.58 (<.0001) |
| Cu Concentration (mg Cu/leaf) | 2.81b | 5.40a | 49.37 (<.0001) |
| Fe Concentration (mg Fe/leaf) | 180.76b | 267.98a | 40.10 (<.0001) |
| Percentage Root Colonization (%) | 56.39a | 30.38b | 461.36 (<.0001) |
| Mycorrhizal Response ratio of root | 0.96 | 1.02 | 1.37 (0.2445) |
