# Supplementary material for: Effect of co-application of phosphorus fertilizer and in vitro-produced mycorrhizal fungal inoculants on yield and leaf nutrient concentration of cassava
Source: PLoS One. 2019 Jun 26;14(6):e0218969. doi: 10.1371/journal.pone.0218969 (PMC6594633; doi:10.1371/journal.pone.0218969)
Supplement: S4 Table — (DOCX) [file pone.0218969.s005.docx]

| Nutrient concentration | Samaru | | | | Minjibir | | | |
| --- | --- | --- | --- | --- | --- | --- | --- | --- |
|  | Root Fresh Weight | Shoot Fresh Weight | Root/Shoot ratio | Root Fresh Weight | | Shoot Fresh Weight | Root/Shoot ratio |  |
| N | 0.641*** | 0.622*** | 0.446*** | 0.033NS | | 0.137NS | 0.104NS |  |
| P | 0.641*** | 0.613*** | 0.458*** | 0.148NS | | 0.279** | 0.245* |  |
| K | 0.603*** | 0.556*** | 0.453*** | 0.20NS | | 0.354** | 0.317** |  |
| Zn | 0.599*** | 0.531*** | 0.451*** | 0.079NS | | 0.250* | 0.197NS |  |
| Cu | 0.469*** | 0.400*** | 0.369*** | 0.133NS | | 0.309** | 0.258* |  |
| Mn | 0.658*** | 0.562*** | 0.517*** | 0.043NS | | 0.203NS | 0.151NS |  |
| Fe | 0.621*** | 0.617*** | 0.440*** | 0.199NS | | 0.365*** | 0.323** |  |

S4 Table
